# Supplementary material for: State of the interactomes: an evaluation of molecular networks for generating biological insights
Source: Mol Syst Biol. 2024 Dec 9;21(1):1–29. doi: 10.1038/s44320-024-00077-y (PMC11697402; doi:10.1038/s44320-024-00077-y)
Supplement: Supplementary file 1 — Appendix [file 44320_2024_77_MOESM1_ESM.pdf]

Appendix for:

State of the Interactomes: an evaluation of molecular networks for generating biological insights.

Sarah N. Wright[1], Scott Colton[1], Leah V. Schaffer[1], Rudolf T. Pillich[1], Christopher Churas[1], Dexter Pratt[1], and Trey Ideker[1,2,*]

[1]Department of Medicine, University of California San Diego, La Jolla, CA 92093, USA
[2]Institute for Genomic Medicine, University of California San Diego, La Jolla, CA 92093, USA
*Correspondence to tideker@ucsd.edu

Table of Contents

Appendix Figure S1. Enrichment for tissue-enhanced gene expression per interactome 2

Appendix Figure S2. Enrichment for tissue-enhanced protein abundance per interactome 3

Appendix Figure S3. AlphaFold-Multimer predictions and presence of experimentally resolved structures from PDB 4

Appendix Figure S4. Optimization of the gene mapping pipeline and gene set recovery parameters 5

Appendix Table S1. Interactome source, version, and citation information 6

Appendix Table S2. Interaction type definitions 8

Appendix Table S3. Source information for experimental gene sets 9

Appendix References 10

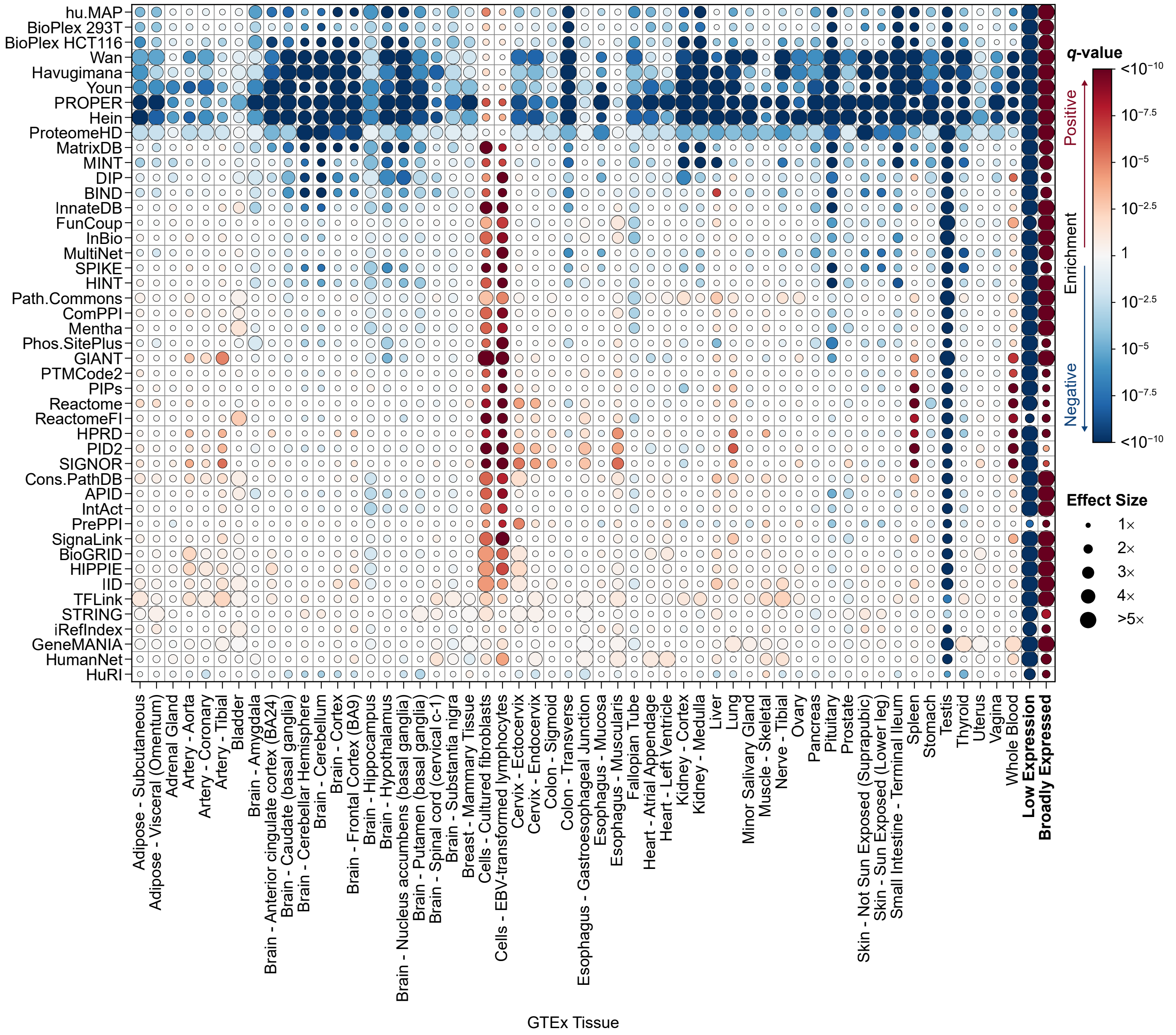

Appendix Figure S1. Enrichment for genes with tissue-enhanced gene expression in each interactome. Color represents the enrichment *q*-value (Fisher's Exact test, BH correction), with blue showing under-enrichment and red showing over-enrichment. The size of the points represents the effect size. Tissue-enhanced gene sets were defined from GTEx to include all genes classified as "Tissue Enriched," "Group Enriched," and "Tissue Enhanced" based on the HPA criteria (Methods). "Low Expression" genes are defined as those with a maximum TPM < 1 across all networks, and all genes not otherwise classified are considered "Broadly Expressed."

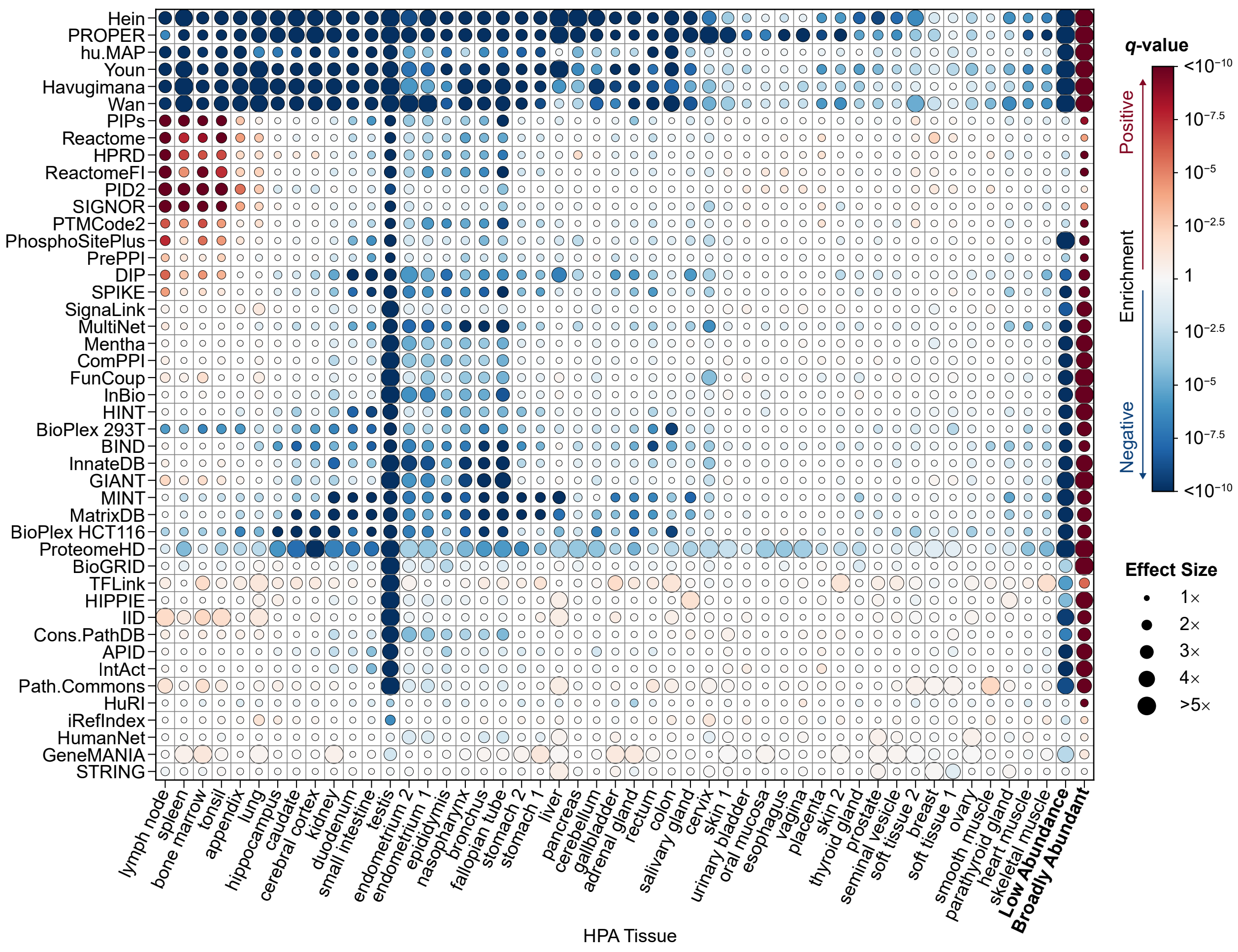

Appendix Figure S2. Enrichment for genes with tissue-enhanced protein abundance in each interactome. Color represents the enrichment *q*-value (Fisher's Exact test, BH correction), with blue showing under-enrichment and red showing over-enrichment. The size of the points represents the effect size. Tissue-enhanced protein sets were defined to include all proteins classified as "Tissue Enriched," "Group Enriched," and "Tissue Enhanced" based on the HPA criteria (Methods). "Low Abundance" proteins were defined as tissues with mean abundance < 0.5, and all proteins not otherwise classified are considered "Broadly Abundant."

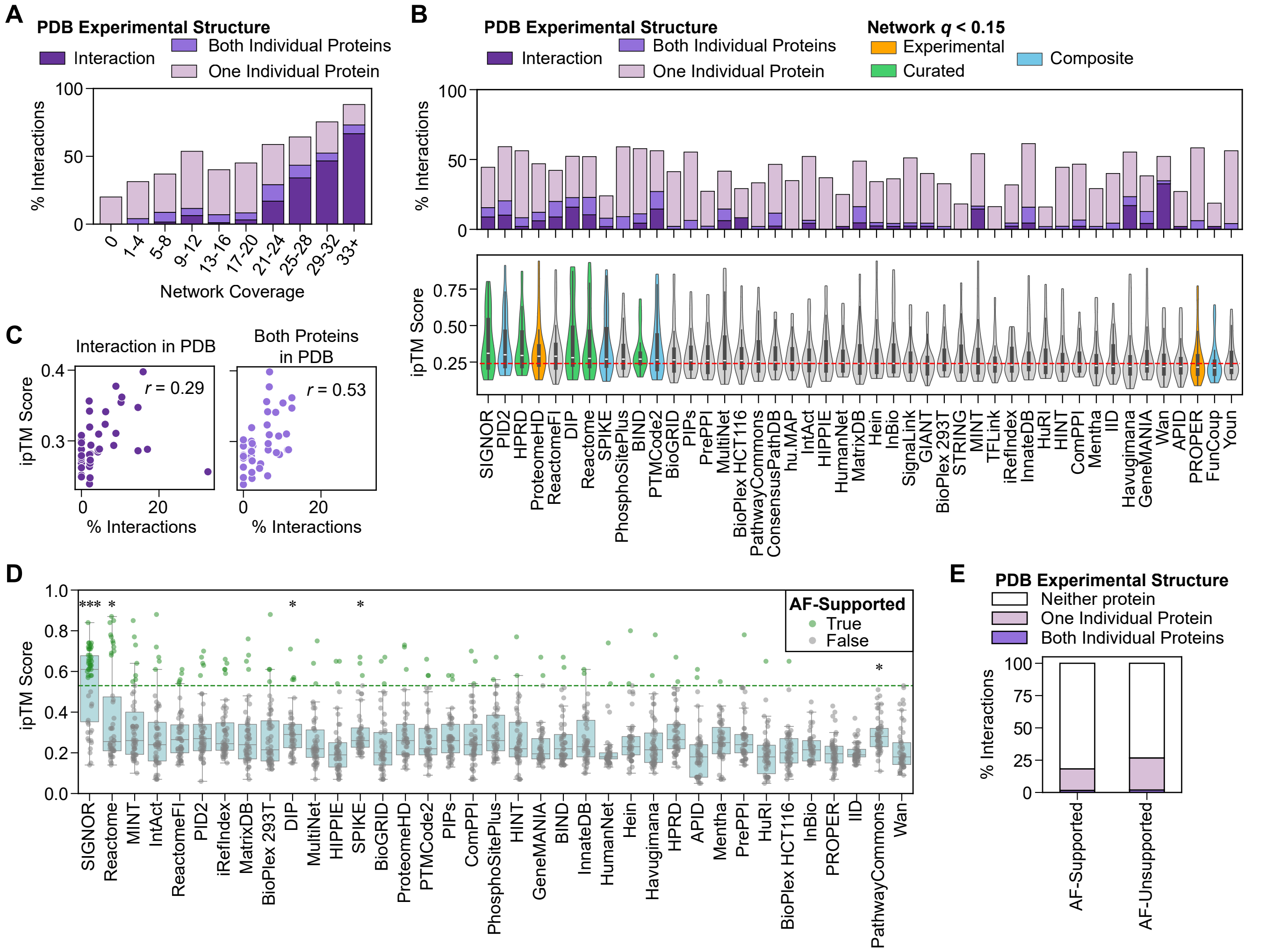

Appendix Figure S3. AlphaFold-Multimer predictions and presence of experimentally resolved structures from PDB. A) Distribution of experimentally resolved structures in PDB as a function of network coverage of interactions. Bars represent 50 interactions per distinct network coverage value. B) Distribution of AlphaFold-Multimer ipTM scores (bottom) and corresponding distribution of experimentally resolved structures in PDB (top) for 50 randomly sampled interactions from each interactome. Colored violins indicate the network has a significantly different ipTM score than 1779 randomly generated protein pairs (Mann-Whitney U-Test, BH correction). Mean ipTM of randomly generated pairs is shown as a red line. Violins extend to the minimum and maximum observations, the center point represents the median, the box represents the interquartile range (Q1 - Q3), and the upper and lower whiskers represent Q1−1.5IQR and Q3+1.5IQR. C) Mean ipTM score per network compared to the fraction of interactions with experimentally resolved structures in PDB. Pearson's correlation reported. D) ipTM scores for previously unreported interactions predicted by each interactome using the MPS algorithm. The green line represents the 95th percentile of ipTM scores (ipTM > 0.53) for 1779 randomly generated protein pairs, with protein pairs above this threshold defined as AF-supported interactions. The distribution of ipTM scores for previously unreported interactions was assessed against the distribution of scores from randomly generated protein pairs by a Mann Whitney U-test (* $q < 0.05$, *** $q < 10^{-5}$, BH correction). The center bar represents the median, the box represents the interquartile range (Q1-Q3), and the upper and lower whiskers represent Q1−1.5IQR and Q3+1.5IQR. E) The distribution of experimentally resolved structures in PDB among previously unreported interactions classified as AF-supported (n = 126) and AF-unsupported (n = 1533). See Dataset EV4 for full results.

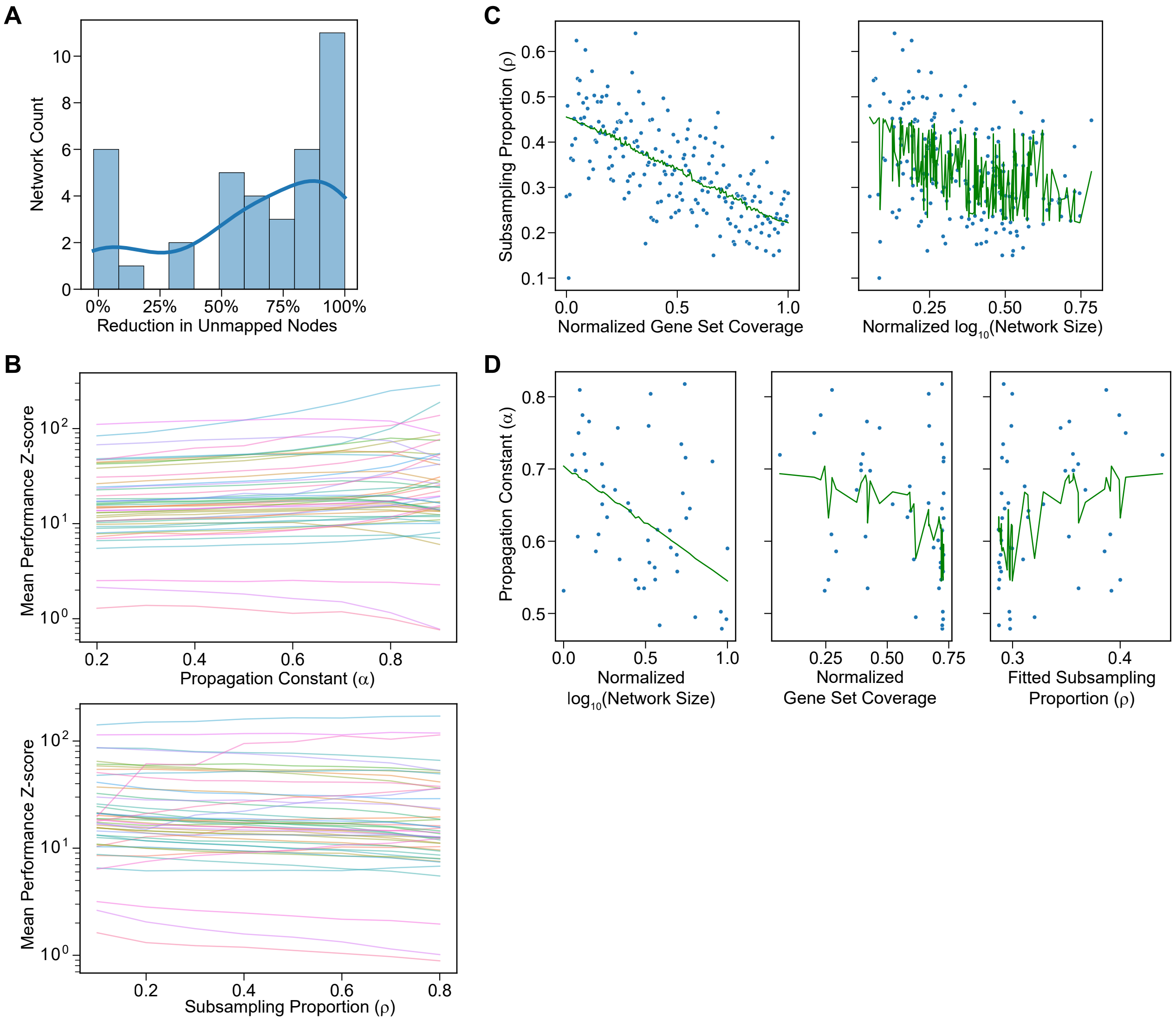

Appendix Figure S4. Optimization of the gene mapping pipeline and gene set recovery parameters. A) Change in the number of unmapped interactors using our updated pipeline compared to MyGeneInfo alone. B) Mean performance Z-score for all interactomes with MSigDB gene sets across a range of propagation constants (top) and subsampling parameters (bottom). Each line represents one interactome. C) Multiple linear regression fit of the optimal subsampling parameter (ρ) to the normalized gene set coverage and normalized network size. D) Multiple linear regression fit of the optimal network propagation constant (α) to the normalized network size, average normalized gene set coverage, and average fitted subsampling parameter (ρ). See Dataset EV6 for full results.

Appendix Table S1. Interactome source, version, and citation information. Where explicit version numbers are not specified by a source, we report the associated year or date of download. Direct download links are provided where possible, otherwise we provide the download options, dataset file name or associated manuscript table.

Name	Version	Source Link	Source Website	Reference(s)
**APID**	2021	http://cicblade.dep.usal.es:8080/APID/init.action#tabr1	http://cicblade.dep.usal.es:8080/APID/init.action	(Alonso-López *et al*, 2016, 2019)
**BIND**	Pathway Commons v12	https://www.pathwaycommons.org/archives/PC2/v12/PathwayCommons12.bind.hgnc.txt.gz	https://www.pathwaycommons.org/pc2/	(Bader *et al*, 2003)
**BioGRID**	227	https://downloads.thebiogrid.org/Download/BioGRID/Release-Archive/BIOGRID-4.4.227/BIOGRID-ORGANISM-4.4.227.tab3.zip	http://thebiogrid.org	(Oughtred *et al*, 2021)
**BioPlex 293T**	3	https://bioplex.hms.harvard.edu/data/BioPlex_293T_Network_10K_Dec_2019.tsv	http://bioplex.hms.harvard.edu	(Huttlin *et al*, 2021)
**BioPlex HCT116**	3	https://bioplex.hms.harvard.edu/data/BioPlex_HCT116_Network_5.5K_Dec_2019.tsv	http://bioplex.hms.harvard.edu	(Huttlin *et al*, 2021)
**ComPPI**	2.1.1	https://comppi.linkgroup.hu/downloads; options: PPI, H. sapiens	https://comppi.linkgroup.hu/	(Veres *et al*, 2015)
**Consensus PathDB**	35	http://cpdb.molgen.mpg.de/download/ConsensusPathDB_human_PPI.gz	http://cpdb.molgen.mpg.de/	(Kamburov *et al*, 2008)
**DIP**	Pathway Commons v12	https://www.pathwaycommons.org/archives/PC2/v12/PathwayCommons12.dip.hgnc.txt.gz	https://www.pathwaycommons.org/pc2/	(Salwinski *et al*, 2004)
**FunCoup**	5	https://funcoup.org/downloads/download.action?type=network&instanceID=24480085&fileName=FC5.0_H.sapiens_full.gz	https://funcoup.org/	(Persson *et al*, 2021)
**GeneMANIA**	2021	http://genemania.org/data/current/Homo_sapiens.COMBINED/COMBINED.DEFAULT_NETWORKS.BP_COMBINING.txt	https://genemania.org/data	(Warde-Farley *et al*, 2010)
**GIANT**	1	https://s3-us-west-2.amazonaws.com/humanbase/networks/global_top.gz	https://hb.flatironinstitute.org/	(Greene *et al*, 2015)
**Havugimana**	1	Table S2	http://human.med.utoronto.ca/	(Havugimana *et al*, 2012)
**Hein**	1	https://www.ebi.ac.uk/intact/search?query=IM-24272&interactorSpeciesFilter=Homo%20sapiens,Mus%20musculus,Saccharomyces%20cerevisiae	https://www.ebi.ac.uk/intact/	(Hein *et al*, 2015)
**HINT**	v4	http://hint.yulab.org/download/	http://hint.yulab.org/	(Das & Yu, 2012)
**HIPPIE**	2.3	http://cbdm-01.zdv.uni-mainz.de/~mschaefer/hippie/HIPPIE-current.mitab.txt	http://cbdm-01.zdv.uni-mainz.de/~mschaefer/hippie/information.php	(Alanis-Lobato *et al*, 2017)
**HPRD**	9	http://hprd.org/download (HPRD_Release9_041310.tar.gz)	http://hprd.org/	(Keshava Prasad *et al*, 2009; Mishra *et al*, 2006; Peri *et al*, 2003)
**HumanNet**	v3	https://staging2.inetbio.org/humannetv3/networks/HumanNet-XC.tsv	https://staging2.inetbio.org/humannetv3/	(Kim *et al*, 2022)
**HumanNet (no CC)**	v3	https://staging2.inetbio.org/humannetv3/networks/HumanNet-FN.tsv	https://staging2.inetbio.org/humannetv3/	(Kim *et al*, 2022)
**hu.MAP**	2	http://humap2.proteincomplexes.org/static/downloads/humap2/humap2_ppis_geneid_20200821.pairsWprob.gz	http://humap2.proteincomplexes.org/	(Drew *et al*, 2021)
**HuRI**	HI-union	http://www.interactome-atlas.org/data/HI-union.tsv	http://www.interactome-atlas.org/	(Luck *et al*, 2020)
**IID**	2021-05	http://iid.ophid.utoronto.ca/static/download/human_annotated_PPIs.txt.gz	http://iid.ophid.utoronto.ca/	(Kotlyar *et al*, 2016)
**InBio**	2016	https://zs-revelen.com/download	https://www.zs-revelen.com/	(Li *et al*, 2017)
**InnateDB**	5.4	https://www.innatedb.com/download/interactions/innatedb_all.mitab.gz	https://www.innatedb.com/	(Lynn *et al*, 2008, 2010; Breuer *et al*, 2013)
**IntAct**	245	https://ftp.ebi.ac.uk/pub/databases/intact/current/psimitab/intact.zip	https://www.ebi.ac.uk/intact/home	(Del Toro *et al*, 2022)

**iRefIndex**	20	https://storage.googleapis.com/irefindex-data/archive/release_20.0/psi_mitab/MITAB2.6/9606.mitab.08-28-2023.txt.zip	https://irefindex.vib.be//	(Razick *et al*, 2008)
**MatrixDB**	2019	http://matrixdb.univ-lyon1.fr/download/matrixdb_FULL.tab.gz	http://matrixdb.univ-lyon1.fr/	(Clerc *et al*, 2019)
**Mentha**	23.11.6	https://mentha.uniroma2.it/dumps/organisms/all.zip	https://mentha.uniroma2.it/index.php	(Calderone *et al*, 2013)
**MINT**	NA	http://www.ebi.ac.uk/Tools/webservices/psicquic/mint/webservices/current/search/query/*	https://mint.bio.uniroma2.it/	(Licata *et al*, 2012)
**MultiNet**	1	http://homes.gersteinlab.org/Khurana-PLoSCompBio-2013/Multinet.interactions.network_presence.txt	http://homes.gersteinlab.org/Khurana-PLoSCompBio-2013/	(Khurana *et al*, 2013)
**Pathway Commons**	12	https://www.pathwaycommons.org/archives/PC2/v12/PathwayCommons12.All.hgnc.txt.gz	pathwaycommons.org	(Rodchenkov *et al*, 2020)
**Phospho SitePlus**	Oct18_23	https://www.phosphosite.org/staticDownloads; Kinase_Substrate_Dataset.gz	https://www.phosphosite.org/	(Hornbeck *et al*, 2015)
**PID**	2	https://www.ndexbio.org/index.html#/networkset/7bc65b82-2a2f-11ed-ac45-0ac135e8bacf	https://www.ndexbio.org/index.html#/	(Pillich *et al*, 2023)
**PIPs**	1	https://www.compbio.dundee.ac.uk/www-pips/dbStats.jsp	https://www.compbio.dundee.ac.uk/www-pips/index.jsp	(McDowall *et al*, 2009)
**PrePPI**	2023	https://honiglab.c2b2.columbia.edu/PrePPI/ref/preppi.human_af.interactome.txt.tar.gz	https://honiglab.c2b2.columbia.edu/PrePPI/	(Petrey *et al*, 2023)
**PROPER**	1	https://genemo.ucsd.edu/proper/PROPER_v1.csv	https://genemo.ucsd.edu/proper/	(Johnson *et al*, 2021)
**ProteomeHD**	1	https://www.proteomehd.net/download_file/S3	https://www.proteomehd.net/index	(Kustatscher *et al*, 2019)
**PTMCode2**	2	https://ptmcode.embl.de/data/PTMcode2_associations_between_proteins.txt.gz	https://ptmcode.embl.de/	(Minguez *et al*, 2015)
**Reactome**	86	https://reactome.org/download/current/interactors/reactome.homo_sapiens.interactions.tab-delimited.txt	https://reactome.org/	(Gillespie *et al*, 2022)
**ReactomeFI**	2022	https://reactome.org/download/tools/ReatomeFIs/FIsInGene_070323_with_annotations.txt.zip	reactome.org	(preprint: Brunson *et al*, 2023)
**SignaLink**	v3.1	http://signalink.org/download	signalink.org	(Csabai *et al*, 2022)
**SIGNOR**	3	https://signor.uniroma2.it/releases/getLatestRelease.php	https://signor.uniroma2.it/	(Lo Surdo *et al*, 2022)
**SPIKE**	1	https://www.cs.tau.ac.il/~spike/download/spikeDB.sif.zip	https://www.cs.tau.ac.il/~spike/	(Paz *et al*, 2011)
**STRING**	12.0	https://stringdb-static.org/download/protein.links.v12.0/9606.protein.links.v12.0.txt.gz	https://string-db.org/	(Szklarczyk *et al*, 2023)
**TFLink**	1	https://cdn.netbiol.org/tflink/download_files/TFLink_Homo_sapiens_interactions_All_mitab_v1.0.tsv.gz	https://tflink.net/	(Liska *et al*, 2022)
**Wan**	1	http://metazoa.med.utoronto.ca/data/High_confidence_16655_correlations_and_ppi_scores.zip	http://metazoa.med.utoronto.ca/	(Wan *et al*, 2015)
**Youn**	1	Supplemental Table 2	http://dx.doi.org/10.1016/j.molcel.2017.12.020	(Youn *et al*, 2018)

Appendix Table S2. Interaction type definitions. Definitions used to define the features of each interactome as in Figure 1A.

Interaction Type	Definition
Physical	Interactions where two biological entities physically come into contact with each other.
Genetic	Interactions where the function or phenotype of one gene is affected by the mutation or absence of another.
Co-Expression	Interactions inferred from the correlated expression patterns of two genes across different conditions or tissues.
Co-Citation	Interactions based on the frequency with which two genes or proteins are mentioned together in scientific literature.
Signaling	Interactions in which one entity sends a signal that is received and processed by another, often leading to a cellular response.
Domain/Structure	Interactions based on the presence of specific protein domains or structural similarity
Metabolic	Interactions within metabolic pathways where the product of one enzyme serves as a substrate for the next
Pathway	Interactions between entities that are part of the same cellular or biochemical pathway
Regulation	Interactions where one entity modulates the activity, stability, or localization of another. This can be positive (activation) or negative (inhibition)
Orthology	Interactions supported by evidence in non-human species, mapped to human genes/proteins via orthology mapping.
Genome	Interactions inferred based on genomic data such as shared regulatory elements, proximity on a chromosome, or shared evolutionary history.
Disease	Interactions derived from evidence suggesting that two genes or proteins are associated with the same disease or phenotype
Functional	Interactions based on shared functional annotations, such as Gene Ontology (GO) terms
Co-Localization	Interactions based on the co-localization of molecules in specific cellular compartments or locations.

Appendix Table S3. Source information for experimental gene sets. Gene set name(s): unique identifiers used throughout gene set analyses. Size: number of distinct genes per set after mapping to NCBI Gene IDs. Experimental Context: brief description of the gene set source publication. Reference(s): primary reference of the gene set. See Dataset EV5 for gene lists.

**Gene set name(s)**	**Size**	**Experimental Context**	**Reference**
Gou24_livercancer<br>*Gou24_lung*	20<br>29	Predictive features from cell-free RNA for early cancer detection and classification.	(Wang *et al*, 2024)
Hashimoto24	166	Differentially expressed genes between responders and non-responders under abatacept treatment for rheumatoid arthritis.	(Iwasaki *et al*, 2024)
Kumbrink24_1v2<br>*Kumbrink24_2v3*	30<br>30	Differentially expressed genes distinguishing low-grade mucinous neoplasia of the appendix, low-grade pseudomyxoma peritonei, and colorectal cancer.	(Pretzsch *et al*, 2024)
Qian24_DEG_PANoptosis	48	Differentially expressed genes of PANoptosis genes in sepsis.	(Dai *et al*, 2023)
Reed24_Catabolic process module<br>*Reed24_Cell cycle module*<br>*Reed24_Cellular response to stimulus module*<br>*Reed24_Endoplasmic reticulum module*<br>*Reed24_Histone demethylation module*<br>*Reed24_Leukocyte module*<br>*Reed24_Neutrophil activation module*<br>*Reed24_Organic acid metabolic process module*	231<br>58<br>45<br>92<br>29<br>75<br>204<br>226	Gene coexpression modules associated with subsets of myeloid cells.	(Sosa *et al*, 2024)
Veleri24_cilia	30	Differentially expressed genes upon knockdown of C2 domain of CC2D2A.	(Jayarajan *et al*, 2024)
Yu24	36	Circulating proteins associated with heart failure development.	(Shah *et al*, 2024)
Zhao24_CAFRGs	43	Prognostic cancer-associated fibroblast-related genes derived from integration of single-cell and bulk transcriptomic data.	(Li *et al*, 2023)

Appendix References

Alanis-Lobato G, Andrade-Navarro MA & Schaefer MH (2017) HIPPIE v2.0: enhancing meaningfulness and reliability of protein-protein interaction networks. *Nucleic Acids Res* 45: D408–D414

Alonso-López D, Campos-Laborie FJ, Gutiérrez MA, Lambourne L, Calderwood MA, Vidal M & De Las Rivas J (2019) APID database: redefining protein-protein interaction experimental evidences and binary interactomes. *Database (Oxford)* 2019

Alonso-López D, Gutiérrez MA, Lopes KP, Prieto C, Santamaría R & De Las Rivas J (2016) APID interactomes: providing proteome-based interactomes with controlled quality for multiple species and derived networks. *Nucleic Acids Res* 44: W529-35

Bader GD, Betel D & Hogue CWV (2003) BIND: The Biomolecular Interaction Network Database. *Nucleic Acids Res* 31: 248–250

Breuer K, Foroushani AK, Laird MR, Chen C, Sribnaia A, Lo R, Winsor GL, Hancock REW, Brinkman FSL & Lynn DJ (2013) InnateDB: systems biology of innate immunity and beyond--recent updates and continuing curation. *Nucleic Acids Res* 41: D1228-33

Brunson T, Sanati N, Matthews L, Haw R, Beavers D, Shorser S, Sevilla C, Viteri G, Conley P, Rothfels K, *et al* (2023) Illuminating Dark Proteins using Reactome Pathways. *bioRxiv* doi: http://dx.doi.org/10.1101/2023.06.05.543335 [PREPRINT]

Calderone A, Castagnoli L & Cesareni G (2013) mentha: a resource for browsing integrated protein-interaction networks. *Nat Methods* 10: 690–691

Clerc O, Deniaud M, Vallet SD, Naba A, Rivet A, Perez S, Thierry-Mieg N & Ricard-Blum S (2019) MatrixDB: integration of new data with a focus on glycosaminoglycan interactions. *Nucleic Acids Res* 47: D376–D381

Csabai L, Fazekas D, Kadlecsik T, Szalay-Bekő M, Bohár B, Madgwick M, Módos D, Ölbei M, Gul L, Sudhakar P, *et al* (2022) SignaLink3: a multi-layered resource to uncover tissue-specific signaling networks. *Nucleic Acids Res* 50: D701–D709

Dai W, Zheng P, Wu J, Chen S, Deng M, Tong X, Liu F, Shang X & Qian K (2023) Integrated analysis of single-cell RNA-seq and chipset data unravels PANoptosis-related genes in sepsis. *Front Immunol* 14: 1247131

Das J & Yu H (2012) HINT: High-quality protein interactomes and their applications in understanding human disease. *BMC Syst Biol* 6: 92

Del Toro N, Shrivastava A, Ragueneau E, Meldal B, Combe C, Barrera E, Perfetto L, How K, Ratan P, Shirodkar G, *et al* (2022) The IntAct database: efficient access to fine-grained molecular interaction data. *Nucleic Acids Res* 50: D648–D653

Drew K, Wallingford JB & Marcotte EM (2021) hu.MAP 2.0: integration of over 15,000 proteomic experiments builds a global compendium of human multiprotein assemblies. *Mol Syst Biol* 17: e10016

Gillespie M, Jassal B, Stephan R, Milacic M, Rothfels K, Senff-Ribeiro A, Griss J, Sevilla C, Matthews L, Gong C, *et al* (2022) The reactome pathway knowledgebase 2022. *Nucleic Acids Res* 50: D687–D692

Greene CS, Krishnan A, Wong AK, Ricciotti E, Zelaya RA, Himmelstein DS, Zhang R, Hartmann BM, Zaslavsky E, Sealfon SC, *et al* (2015) Understanding multicellular function and disease with human tissue-specific networks. *Nat Genet* 47: 569–576

Havugimana PC, Hart GT, Nepusz T, Yang H, Turinsky AL, Li Z, Wang PI, Boutz DR, Fong V, Phanse S, *et al* (2012) A census of human soluble protein complexes. *Cell* 150: 1068–1081

Hein MY, Hubner NC, Poser I, Cox J, Nagaraj N, Toyoda Y, Gak IA, Weisswange I, Mansfeld J, Buchholz F, *et al* (2015) A human interactome in three quantitative dimensions organized by stoichiometries and abundances. *Cell* 163: 712–723

Hornbeck PV, Zhang B, Murray B, Kornhauser JM, Latham V & Skrzypek E (2015) PhosphoSitePlus, 2014: mutations, PTMs and recalibrations. *Nucleic Acids Res* 43: D512-20

Huttlin EL, Bruckner RJ, Navarrete-Perea J, Cannon JR, Baltier K, Gebreab F, Gygi MP, Thornock A, Zarraga G, Tam S, *et al* (2021) Dual proteome-scale networks reveal cell-specific remodeling of the human interactome. *Cell* 184: 3022-3040.e28

Iwasaki T, Watanabe R, Ito H, Fujii T, Ohmura K, Yoshitomi H, Murata K, Murakami K, Onishi A, Tanaka M, *et al* (2024) Monocyte-derived transcriptomes explain the ineffectiveness of abatacept in rheumatoid arthritis. *Arthritis Res Ther* 26: 1

Jayarajan RO, Chakraborty S, Raghu KG, Purushothaman J & Veleri S (2024) Joubert syndrome causing mutation in C2 domain of CC2D2A affects structural integrity of cilia and cellular signaling molecules. *Exp Brain Res*

Johnson KL, Qi Z, Yan Z, Wen X, Nguyen TC, Zaleta-Rivera K, Chen C-J, Fan X, Sriram K, Wan X, *et al* (2021) Revealing protein-protein interactions at the transcriptome scale by sequencing. *Mol Cell* 81: 4091-4103.e9

Kamburov A, Wierling C, Lehrach H & Herwig R (2008) ConsensusPathDB—a database for integrating human functional interaction networks. *Nucleic Acids Res* 37: D623–D628

Keshava Prasad TS, Goel R, Kandasamy K, Keerthikumar S, Kumar S, Mathivanan S, Telikicherla D, Raju R, Shafreen B, Venugopal A, *et al* (2009) Human Protein Reference Database--2009 update. *Nucleic Acids Res* 37: D767-72

Khurana E, Fu Y, Chen J & Gerstein M (2013) Interpretation of genomic variants using a unified biological network approach. *PLoS Comput Biol* 9: e1002886

Kim CY, Baek S, Cha J, Yang S, Kim E, Marcotte EM, Hart T & Lee I (2022) HumanNet v3: an improved database of human gene networks for disease research. *Nucleic Acids Res* 50: D632–D639

Kotlyar M, Pastrello C, Sheahan N & Jurisica I (2016) Integrated interactions database: tissue-specific view of the human and model organism interactomes. *Nucleic Acids Res* 44: D536-41

Kustatscher G, Grabowski P, Schrader TA, Passmore JB, Schrader M & Rappsilber J (2019) Co-regulation map of the human proteome enables identification of protein functions. *Nat Biotechnol* 37: 1361–1371

Li C, Yang L, Zhang Y, Hou Q, Wang S, Lu S, Tao Y, Hu W & Zhao L (2023) Integrating single-cell and bulk transcriptomic analyses to develop a cancer-associated fibroblast-derived biomarker for predicting prognosis and therapeutic response in breast cancer. *Front Immunol* 14: 1307588

Li T, Wernersson R, Hansen RB, Horn H, Mercer J, Slodkowicz G, Workman CT, Rigina O, Rapacki K, Stærfeldt HH, *et al* (2017) A scored human protein-protein interaction network to catalyze genomic interpretation. *Nat Methods* 14: 61–64

Licata L, Briganti L, Peluso D, Perfetto L, Iannuccelli M, Galeota E, Sacco F, Palma A, Nardozza AP, Santonico E, *et al* (2012) MINT, the molecular interaction database: 2012 update. *Nucleic Acids Res* 40: D857-61

Liska O, Bohár B, Hidas A, Korcsmáros T, Papp B, Fazekas D & Ari E (2022) TFLink: an integrated gateway to access transcription factor-target gene interactions for multiple species. *Database* 2022

Lo Surdo P, Iannuccelli M, Contino S, Castagnoli L, Licata L, Cesareni G & Perfetto L (2022) SIGNOR 3.0, the SIGnaling network open resource 3.0: 2022 update. *Nucleic Acids Res* 51: D631–D637

Luck K, Kim D-K, Lambourne L, Spirohn K, Begg BE, Bian W, Brignall R, Cafarelli T, Campos-Laborie FJ, Charloteaux B, *et al* (2020) A reference map of the human binary protein interactome. *Nature* 580: 402–408

Lynn DJ, Chan C, Naseer M, Yau M, Lo R, Sribnaia A, Ring G, Que J, Wee K, Winsor GL, *et al* (2010) Curating the innate immunity interactome. *BMC Syst Biol* 4: 117

Lynn DJ, Winsor GL, Chan C, Richard N, Laird MR, Barsky A, Gardy JL, Roche FM, Chan THW, Shah N, *et al* (2008) InnateDB: facilitating systems-level analyses of the mammalian innate immune response. *Mol Syst Biol* 4: 218

McDowall MD, Scott MS & Barton GJ (2009) PIPs: human protein-protein interaction prediction database. *Nucleic Acids Res* 37: D651-6

Minguez P, Letunic I, Parca L, Garcia-Alonso L, Dopazo J, Huerta-Cepas J & Bork P (2015) PTMcode v2: a resource for functional associations of post-translational modifications within and between proteins. *Nucleic Acids Res* 43: D494-502

Mishra GR, Suresh M, Kumaran K, Kannabiran N, Suresh S, Bala P, Shivakumar K, Anuradha N, Reddy R, Raghavan TM, *et al* (2006) Human protein reference database--2006 update. *Nucleic Acids Res* 34: D411-4

Oughtred R, Rust J, Chang C, Breitkreutz B-J, Stark C, Willems A, Boucher L, Leung G, Kolas N, Zhang F, *et al* (2021) The BioGRID database: A comprehensive biomedical resource of curated protein, genetic, and chemical interactions. *Protein Sci* 30: 187–200

Paz A, Brownstein Z, Ber Y, Bialik S, David E, Sagir D, Ulitsky I, Elkon R, Kimchi A, Avraham KB, *et al* (2011) SPIKE: a database of highly curated human signaling pathways. *Nucleic Acids Res* 39: D793-9

Peri S, Navarro JD, Amanchy R, Kristiansen TZ, Jonnalagadda CK, Surendranath V, Niranjan V, Muthusamy B, Gandhi TKB, Gronborg M, *et al* (2003) Development of human protein reference database as an initial platform for approaching systems biology in humans. *Genome Res* 13: 2363–2371

Persson E, Castresana-Aguirre M, Buzzao D, Guala D & Sonnhammer ELL (2021) FunCoup 5: Functional Association Networks in All Domains of Life, Supporting Directed Links and Tissue-Specificity. *J Mol Biol* 433: 166835

Petrey D, Zhao H, Trudeau SJ, Murray D & Honig B (2023) PrePPI: A structure informed proteome-wide database of protein-Protein Interactions. *J Mol Biol* 435: 168052

Pillich RT, Chen J, Churas C, Fong D, Gyori BM, Ideker T, Karis K, Liu SN, Ono K, Pico A, *et al* (2023) NDEx IQuery: a multi-method network gene set analysis leveraging the Network Data Exchange. *Bioinformatics* 39

Pretzsch E, Neumann J, Nieß H, Pretzsch CM, Hofmann FO, Kirchner T, Klauschen F, Werner J, Angele M & Kumbrink J (2024) Comparative transcriptomic analyses reveal activation of the epithelial-mesenchymal transition program in non-metastasizing low grade pseudomyxoma peritonei. *Pathol Res Pract* 254: 155129

Razick S, Magklaras G & Donaldson IM (2008) iRefIndex: a consolidated protein interaction database with provenance. *BMC Bioinformatics* 9: 405

Rodchenkov I, Babur O, Luna A, Aksoy BA, Wong JV, Fong D, Franz M, Siper MC, Cheung M, Wrana M, *et al* (2020) Pathway Commons 2019 Update: integration, analysis and exploration of pathway data. *Nucleic Acids Res* 48: D489–D497

Salwinski L, Miller CS, Smith AJ, Pettit FK, Bowie JU & Eisenberg D (2004) The Database of Interacting Proteins: 2004 update. *Nucleic Acids Res* 32: D449-51

Shah AM, Myhre PL, Arthur V, Dorbala P, Rasheed H, Buckley LF, Claggett B, Liu G, Ma J, Nguyen NQ, *et al* (2024) Large scale plasma proteomics identifies novel proteins and protein networks associated with heart failure development. *Nat Commun* 15: 528

Sosa RA, Ahn R, Li F, Terry AQ, Qian Z, Bhat A, Sen S, Naini BV, Ito T, Kaldas FM, *et al* (2024) Myeloid spatial and transcriptional molecular signature of ischemia-reperfusion injury in human liver transplantation. *Hepatol Commun* 8

Szklarczyk D, Kirsch R, Koutrouli M, Nastou K, Mehryary F, Hachilif R, Gable AL, Fang T, Doncheva NT, Pyysalo S, *et al* (2023) The STRING database in 2023: protein-protein association networks and functional enrichment analyses for any sequenced genome of interest. *Nucleic Acids Res* 51: D638–D646

Veres DV, Gyurkó DM, Thaler B, Szalay KZ, Fazekas D, Korcsmáros T & Csermely P (2015) ComPPI: a cellular compartment-specific database for protein-protein interaction network analysis. *Nucleic Acids Res* 43: D485-93

Wan C, Borgeson B, Phanse S, Tu F, Drew K, Clark G, Xiong X, Kagan O, Kwan J, Bezginov A, *et al* (2015) Panorama of ancient metazoan macromolecular complexes. *Nature* 525: 339–344

Wang J, Huang J, Hu Y, Guo Q, Zhang S, Tian J, Niu Y, Ji L, Xu Y, Tang P, *et al* (2024) Terminal modifications independent cell-free RNA sequencing enables sensitive early cancer detection and classification. *Nat Commun* 15: 156

Warde-Farley D, Donaldson SL, Comes O, Zuberi K, Badrawi R, Chao P, Franz M, Grouios C, Kazi F, Lopes CT, *et al* (2010) The GeneMANIA prediction server: biological network integration for gene prioritization and predicting gene function. *Nucleic Acids Res* 38: W214-20

Youn J-Y, Dunham WH, Hong SJ, Knight JDR, Bashkurov M, Chen GI, Bagci H, Rathod B, MacLeod G, Eng SWM, *et al* (2018) High-Density Proximity Mapping Reveals the Subcellular Organization of mRNA-Associated Granules and Bodies. *Mol Cell* 69: 517-532.e11
