# Supplementary material for: State of the interactomes: an evaluation of molecular networks for generating biological insights
Source: Mol Syst Biol. 2024 Dec 9;21(1):1–29. doi: 10.1038/s44320-024-00077-y (PMC11697402; doi:10.1038/s44320-024-00077-y)
Supplement: Supplementary file 9 — Expanded View Figures [file 44320_2024_77_MOESM9_ESM.pdf]

Expanded View Figures

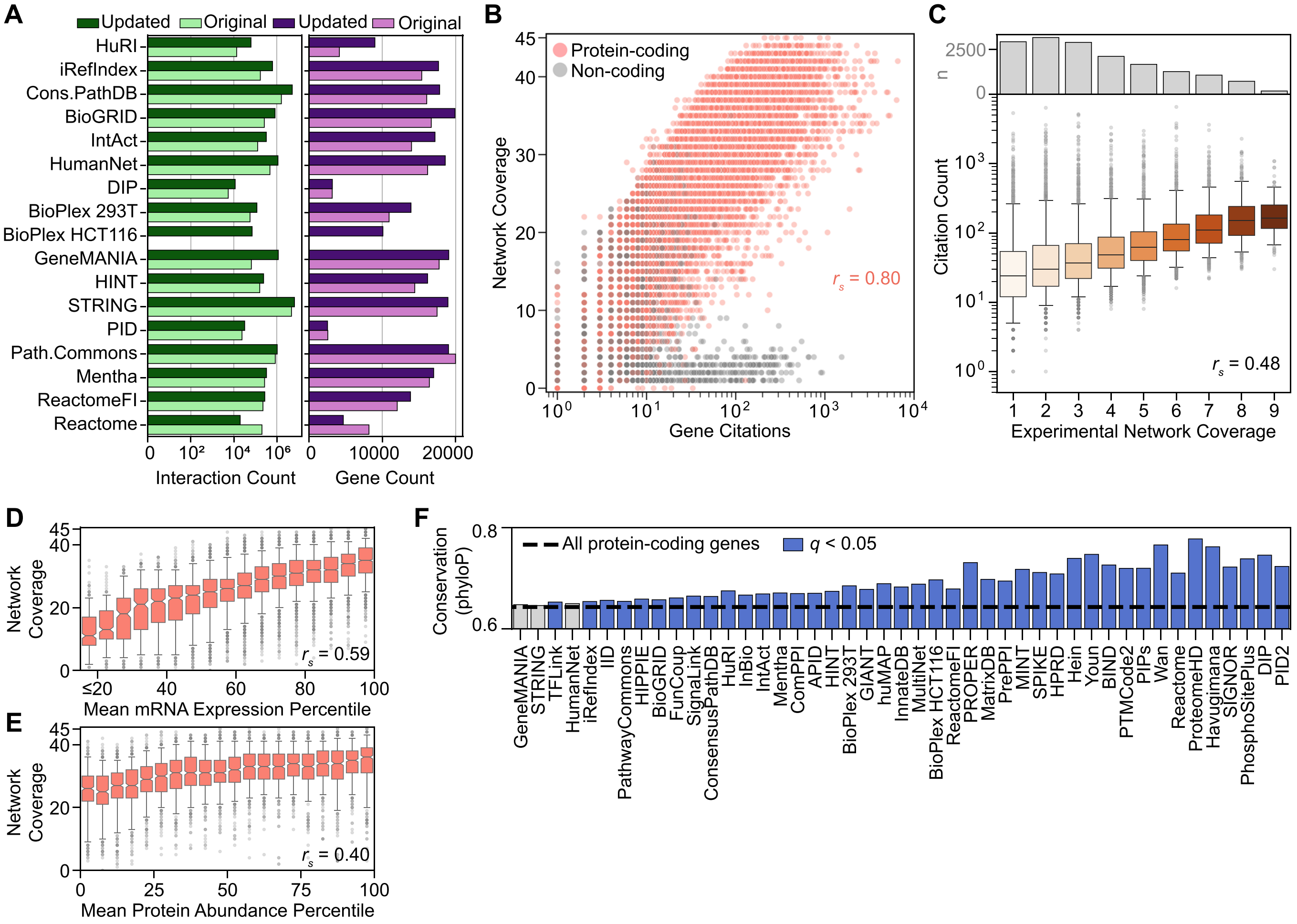

Figure EV1. Global patterns in interactome gene representation.

(**A**) Interaction and gene counts for networks updated between Huang et al (2018) and the present study. Updated networks are those where the corresponding database has been updated or where our source of the network was changed. Values represent distinct genes and interactions after data processing and mapping of identifiers to NCBI Gene IDs. (**B**) Comparison of network coverage and NCBI gene citation count as of December 20, 2023, for genes that are protein-coding or present in at least one interactome. Genes with no reported citations are excluded. Spearman correlation reported for protein-coding genes. (**C**) Box plots of citation count for protein-coding genes with at least one citation and network coverage in the nine experimental interactomes. Top bar plot shows the number of distinct interactions per network coverage value. The center of each box plot represents the median, the box boundaries correspond to the upper and lower quartiles, and the whiskers extend to the 5th and 95th percentiles. Spearman correlation reported. (**D**, **E**) Network coverage of protein-coding genes as a function of mean mRNA expression across all tissues in GTEx (**D**) and mean protein abundance across all tissues in the Human Protein Atlas (HPA) (**E**). The center of each box plot represents the median, the box boundaries correspond to the upper and lower quartiles, and the whiskers extend to the 5th and 95th percentiles. Spearman correlations reported. In (**D**), each box represents 1740 genes, except for the ≤20th percentile box which represents 6963 genes. In (**E**), each box represents between 520 and 596 genes (median 557 genes). (**F**) Median gene conservation scores for protein-coding genes in each interactome, compared to the median of all protein-coding genes (black line). Colored bars show networks with significantly higher median conservation scores than the baseline of all protein-coding genes (permutation test, $q < 0.05$, Bonferroni correction). Networks plotted in the same order as in Fig. 2C.

© The Author(s)

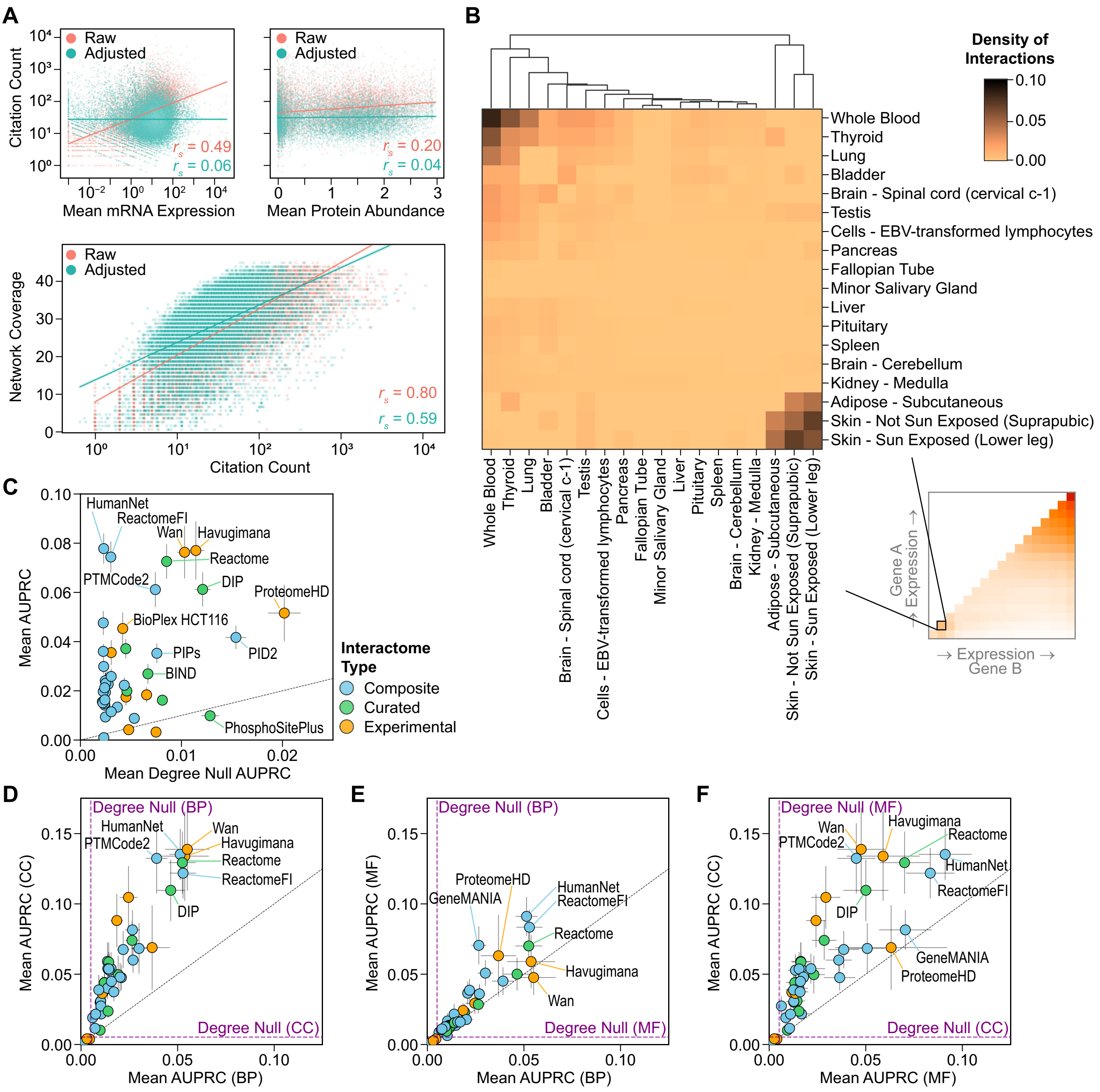

Figure EV2. Expanded analysis of interactome biases and physiological gene function predictions.

(**A**) Correlations between citation count and mRNA expression, protein abundance and network coverage for protein-coding genes. Original citation counts ("Raw"), and citation counts adjusted for mean gene mRNA expression using log–log ordinary least squares regression ("Adjusted"). Spearman correlations reported. (**B**) Interaction density of genes from the 20–25th percentile of mean mRNA expression across select tissues. Genes were filtered to those with non-zero expression in a maximum of two tissues (1400/1740 genes). Tissues with at least one reported interaction amongst the filtered genes are shown and clustered by minimum distance. The inset shows the corresponding bin location in Fig. 2F. (**C–F**) Gene Ontology (GO) gene function prediction via neighbor-voting. The area under the precision-recall curve (AUPRC) was calculated using 5-fold cross-validation with error bars indicating 95% confidence intervals, with mean taken across all GO annotations tested. Points colored by network classification. (**C**) Mean AUPRC across all GO annotations tested compared to mean Null AUPRC calculated from gene degree alone. The specific number of GO terms evaluated per network ranged from 268 to 1459 (median 1301) terms. See Dataset EV1 for full details. (**D–F**) Mean AUPRC for GO annotations within each GO branch (BP: biological process, CC: cellular component, MF: molecular function). Purple dashed lines show the mean null AUPRC from node degree alone across all interactomes. Black dashed lines are the identity lines. The specific number of GO terms evaluated per network per branch ranged from BP: 133 to 843 (median 731) terms, CC: 71 to 317 (median 295) terms, MF: 64 to 301 (median 278) terms. See Dataset EV1 for full details.

© The Author(s)

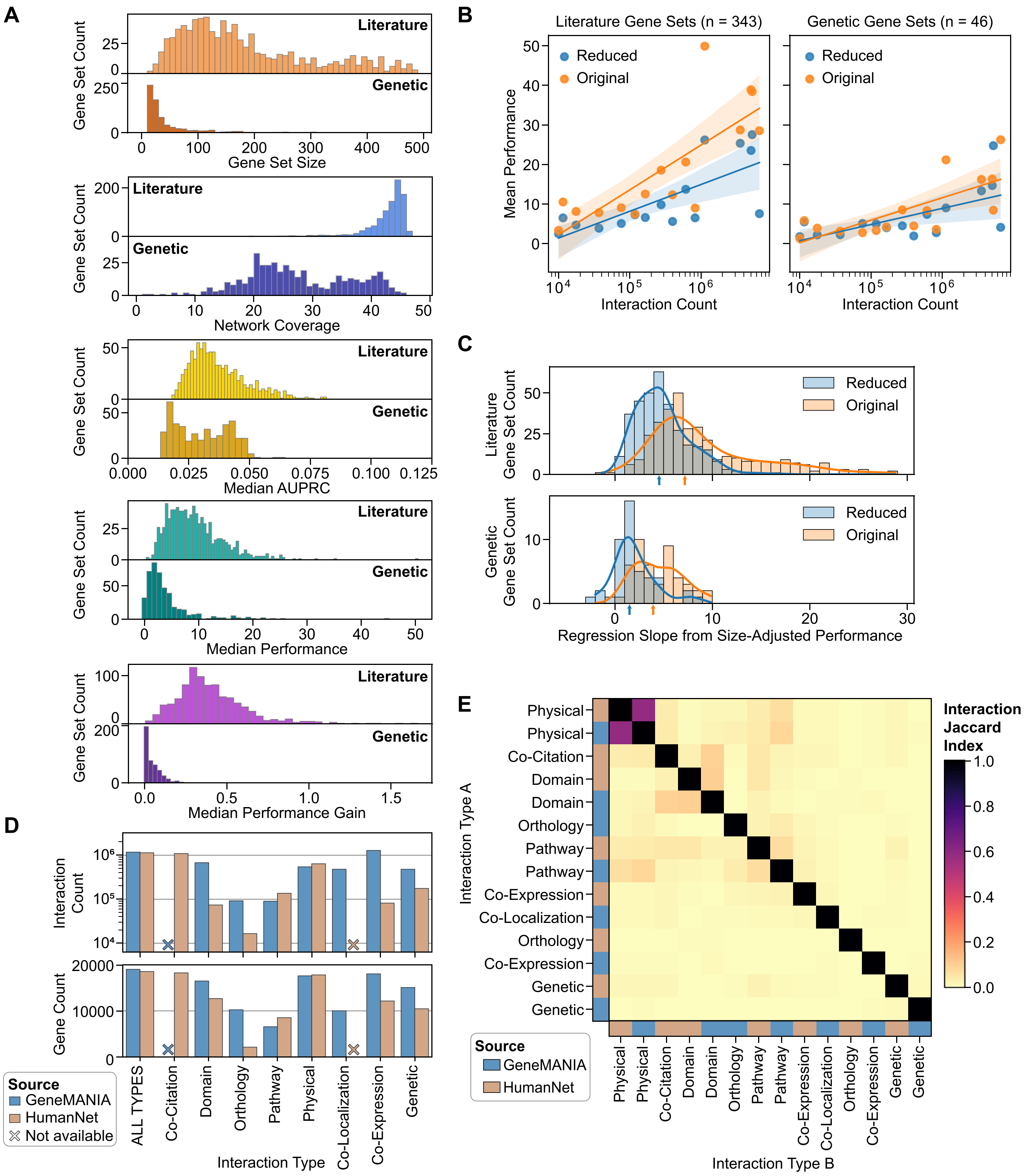
A
Gene Set Count
Literature
Genetic
Gene Set Size
Network Coverage
Median AUPRC
Median Performance
Median Performance Gain
B
Literature Gene Sets (n = 343)
Genetic Gene Sets (n = 46)
Mean Performance
Reduced
Original
Interaction Count
C
Literature Gene Set Count
Genetic Gene Set Count
Reduced
Original
Regression Slope from Size-Adjusted Performance
D
Interaction Count
Gene Count
Source
GeneMANIA
HumanNet
Not available
ALL TYPES
Co-Citation
Domain
Orthology
Pathway
Physical
Co-Localization
Co-Expression
Genetic
Interaction Type
E
Interaction Jaccard Index
Interaction Type A
Interaction Type B
Physical
Physical
Co-Citation
Domain
Domain
Orthology
Pathway
Pathway
Co-Expression
Co-Localization
Orthology
Co-Expression
Genetic
Genetic
Source
GeneMANIA
HumanNet

Figure EV3. Descriptive statistics of gene set recovery performance and type-specific networks.

(**A**) Distributions of gene set statistics and gene set recovery performance metrics for 45 interactomes. Gene set size: number of unique genes in each set after mapping to NCBI Gene IDs. Network Coverage: number of networks containing with at least 20 set genes. AUPRC, Performance, Performance Gain metrics: median metric of a gene set across all networks containing at least 20 set genes. See Dataset EV2 for full gene set recovery results and Dataset EV5 for full gene lists. (**B**, **C**) Gene set recovery performance for a subset of 16 interactomes, controlling for gene set coverage. Given a set of disease-associated genes, the "original" set represents the genes present in each interactome independently, while the "reduced" set represents the maximal subset of genes present in all 16 interactomes. (**B**) Mean gene set recovery performance with Literature and Genetic gene sets for each interactome relative to interactome size (interaction count). The lines show log-linear fits with 95% confidence intervals. See also Dataset EV2. (**C**) Distribution of regression slopes between interactome size and gene set recovery performance for Literature and Genetic gene sets, as calculated for size-adjusted performance. Arrows indicate the median regression slopes. (**D**) Sizes of interaction-type-specific interactomes defined from HumanNet and GeneMANIA. Crosses indicate that a network could not be defined from available data. Interaction type 'ALL TYPES' refers to the HumanNet and GeneMANIA networks used in the primary analysis, which include all types of interactions. (**E**) Interaction similarities of interaction-type-specific networks. Similarities measured by the Jaccard Index of network interactions, and clustered using the Ward variance minimization algorithm.

 © The Author(s)

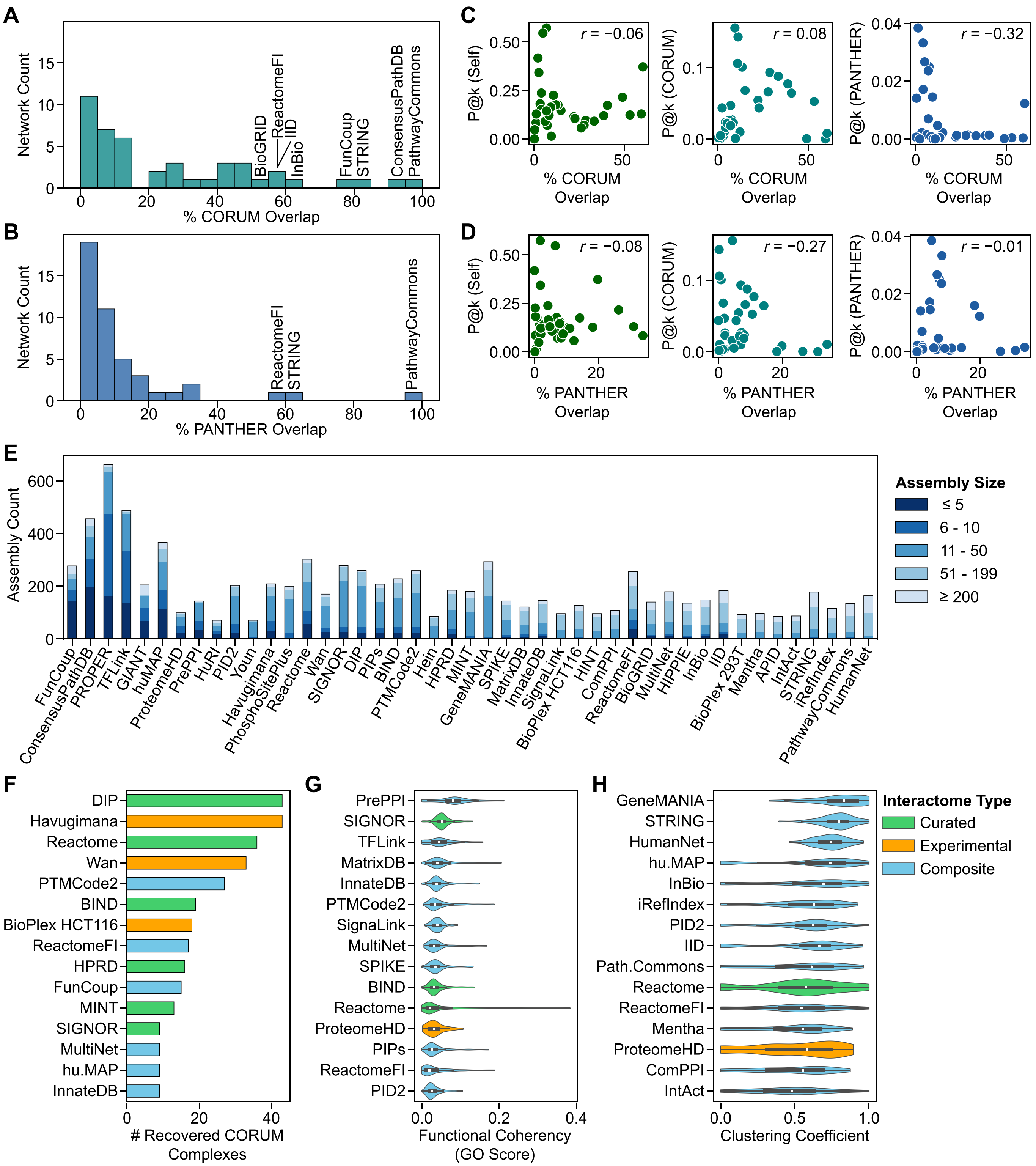
A
Network Count
% CORUM Overlap
BioGRID
ReactomeFI
IID
InBio
FunCoup
STRING
ConsensusPathDB
PathwayCommons
B
Network Count
% PANTHER Overlap
ReactomeFI
STRING
PathwayCommons
C
P@k (Self)
P@k (CORUM)
P@k (PANTHER)
% CORUM Overlap
r = −0.06
r = 0.08
r = −0.32
D
% PANTHER Overlap
r = −0.08
r = −0.27
r = −0.01
E
Assembly Count
Assembly Size
≤ 5
6 - 10
11 - 50
51 - 199
≥ 200
FunCoup
ConsensusPathDB
PROPER
TFLink
GIANT
huMAP
ProteomeHD
PrePPI
HuRI
PID2
Youn
Havugimana
PhosphoSitePlus
Reactome
Wan
SIGNOR
DIP
PIPs
BIND
PTMCode2
Hein
HPRD
MINT
GeneMANIA
SPIKE
MatrixDB
InnateDB
SignaLink
BioPlex HCT116
HINT
CompPPI
ReactomeFI
BioGRID
MultiNet
HIPPIE
InBio
IID
BioPlex 293T
Mentha
APID
IntAct
STRING
iRefIndex
PathwayCommons
HumanNet
F
DIP
Havugimana
Reactome
Wan
PTMCode2
BIND
BioPlex HCT116
ReactomeFI
HPRD
FunCoup
MINT
SIGNOR
MultiNet
hu.MAP
InnateDB
Recovered CORUM Complexes
G
PrePPI
SIGNOR
TFLink
MatrixDB
InnateDB
PTMCode2
SignaLink
MultiNet
SPIKE
BIND
Reactome
ProteomeHD
PIPs
ReactomeFI
PID2
Functional Coherency (GO Score)
H
GeneMANIA
STRING
HumanNet
hu.MAP
InBio
iRefIndex
PID2
IID
Path.Commons
Reactome
ReactomeFI
Mentha
ProteomeHD
ComPPI
IntAct
Clustering Coefficient
Interactome Type
Curated
Experimental
Composite

Figure EV4. Overlap with external complex and pathway interaction sets and quality of predicted complexes.

(**A**, **B**) Distribution of the fraction of CORUM (**A**) and PANTHER (**B**) interactions present in each interactome. Interactomes with the highest overlap are labeled. (**C**, **D**) Interaction prediction performance (mean of P@k using the MPS algorithm) as a function of interaction overlap with CORUM (**C**) and PANTHER (**D**), with associated Pearson's correlation value. (**E**) Size distributions of protein assemblies detected via hierarchical community detection for each interactome, sorted by median assembly size. (**F–H**) Evaluation of protein assemblies predicted by hierarchical community detection. The 15 top-performing networks are shown for each metric and colored by interactome classification. (**F**) Number of CORUM complexes recovered, defined as the number of CORUM complexes with a Jaccard similarity of ≥0.5 with any predicted assembly. (**G**) Functional coherency of predicted assemblies with fewer than 200 proteins based on the mean semantic similarity of GO annotations between assembly proteins (Methods). (**H**) Distribution of clustering coefficients of predicted assemblies with fewer than 200 proteins. In (**G**, **H**), the violins extend to the minimum and maximum observations, and represent the number of complexes with fewer than 200 genes per network as displayed in (**E**). The center bar represents the median, the box represents the interquartile range (Q1–Q3), and the upper and lower whiskers represent Q1 − 1.5IQR and Q3 + 1.5IQR.

© The Author(s)

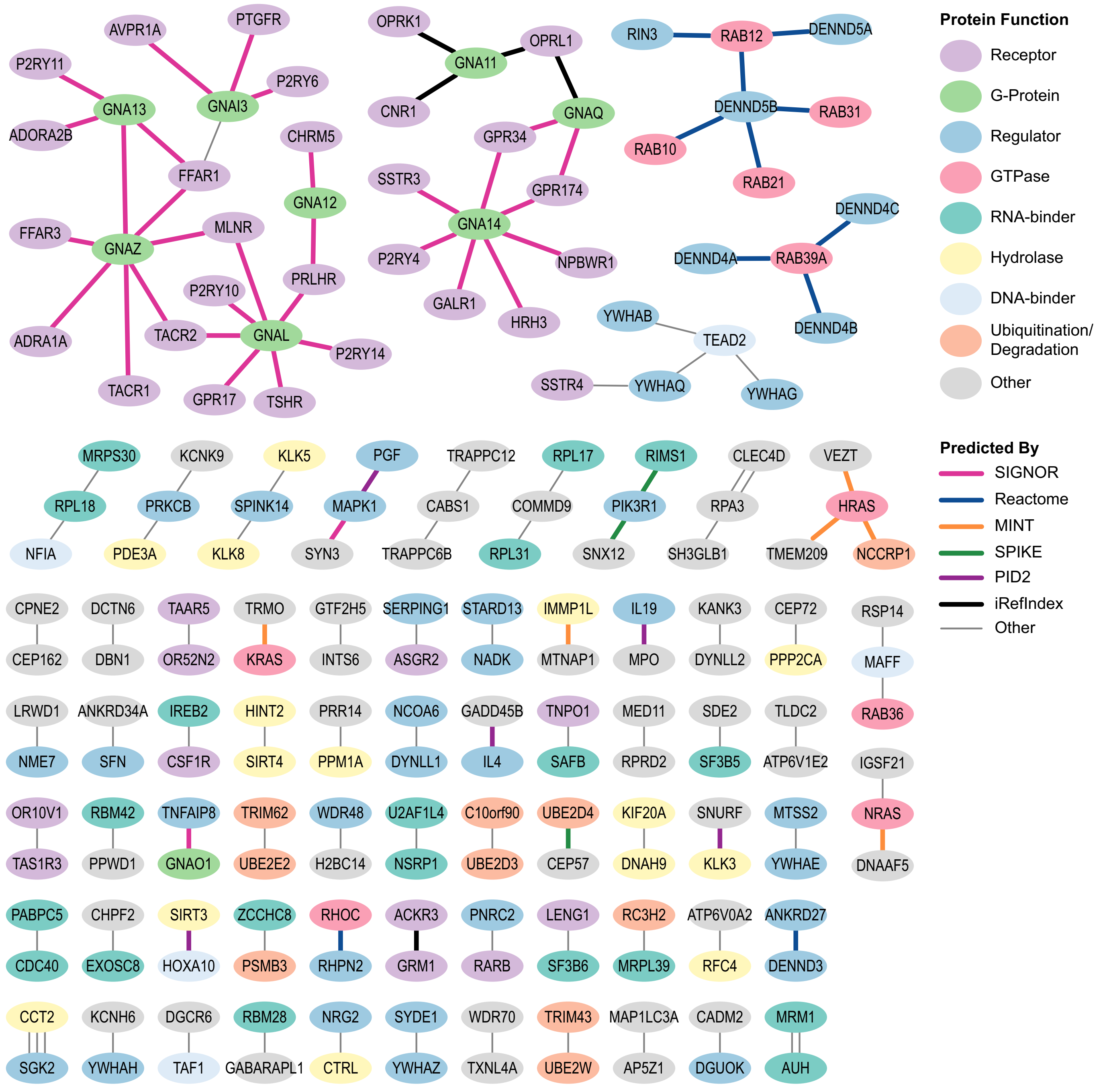

Figure EV5. Subnetwork of all previously unreported interactions classified as AF-supported.

Node color indicates broad protein function and edge color indicates the predicting interactome. Protein pairs linked by multiple edges were predicted in the top 50 previously unreported interactions by multiple networks.

© The Author(s)
